# Effect of ascorbic acid on cellular respiration with mitochondrial reductase in gingival fibroblast

**DOI:** 10.6026/97320630019552

**Published:** 2023-05-31

**Authors:** Chanchal Katariya, ND Jayakumar, Raghunandha Kumar

**Affiliations:** 1 Department of Periodontics, Saveetha Dental College, Saveetha Institute of Medical and Technical Sciences (SIMATS), Saveetha University, Chennai, India

**Keywords:** Ascorbic acid, gingival fibroblast, mitochondrial reductase

## Abstract

Vitamin C or L-ascorbic acid has diverse functions in the body, especially healing promotion in tissue injury via participating in the hydroxylation reactions required for collagen formation. Systemic administration of vitamin C plays an important role on gingival fibroblast proliferation and functions. Whether local or rinsing administration of vitamin C alters gingival fibroblast wound healing behavior remains unclear. Therefore, it is of interest to investigate the effect of vitamin C on gingival fibroblast behavior utilizing cell culture. Primary human gingival fibroblasts isolated from gingival tissue were rinsed with medium containing various concentrations of vitamin C. Cell migration, cell viability was assessed using MTT assay. The viability assay showed >95% live cells, and no significant (P > 0.05) difference in these values was observed at different concentrations at 24 hrs. The levels of cell proliferation were not significantly different among the control and experimental groups in 24 hrs experimental time-points (p > 0.05). Vitamin C is nontoxic and can be used for further experiments to validate for clinical application. This was further confirmed with morphological examination after 24hrs incubation on control and experimental group drugs observed under the phase contrast microscope.

## Background:

Gingival fibroblasts are one of the primary cells involved in periodontal regeneration. Gingival fibroblasts are responsible for In addition to their traditional function in the production of ATP[1,2], mitochondria also house a number of additional metabolic pathways, including the oxidation of fatty acids, the synthesis of iron-sulfur clusters, and oxygen metabolism. Additionally, mitochondria play a crucial role in a number of regulatory mechanisms, such as stress-related or developmental cell death. The primary method for producing ATP in eukaryotes is mitochondrial oxidative phosphorylation (ox-phos).[3] Through a series of respiratory H+ pumps, electrons released from reducing substrates are transferred to O2 in this process. These pumps (complexes I-IV) create an electrochemical gradient of H+ across the inner mitochondrial membrane, which is then used to power complex V's synthesis of ATP - ATP synthase.[4] Chemically, the progressive reduction of O2 occurs via a number of reactive oxygen species (O2 → O2 → H2O2 → OH → H2O). These ROS have the potential to harm DNA, proteins, and lipids in cells. [5] Mitochondrial ox-phos performs the delicate balancing act of aerobic metabolism, reducing O2 to H2O while increasing ATP synthesis and limiting ROS generation to only the levels necessary for micro-domain cell signaling. [6,7] In addition to being harmful byproducts of respiration, mitochondrial ROS are crucial for cell signaling. [8] In programmed cell death, the release of substances from the mitochondria, such as cytochrome c, is a crucial phase. [9,10] In the past, ROS were believed to be detrimental byproducts of respiration that caused oxidative damage and accelerated ageing. However, new research has revealed that ROS produced from mitochondria are crucial for a variety of cell signaling activities. [8] Studies show that vitamins have proven to have a protective role against oxidative stress in the body. Dietary supplementation with supra nutritional doses of the antioxidant vitamins C and E protected against the signs of mitochondrial oxidative stress like lipid peroxidation and an oxidation of glutathione. [11] Vitamin C or ascorbic acid, a unique vitamin which exerts a reducing and antioxidant effect, scavenger for free radicals, and most importantly acts as an enzyme cofactor in collagen synthesis. [12] It is said to have astounding wound healing properties. Vitamin C is considered an essential dietary oxidant for periodontal health since it scavenges excessive ROS [13] Vitamin C serves as a cofactor for the enzymes prolysyl and lysyl hydroxylase, the enzymes that are responsible for stabilizing and cross-linking the collagen molecules. Vitamin C acts as an antioxidant, by acting as an electron donor Thereby preventing lipid peroxidation by ROS. [14] Vitamin C also controls collagen synthesis by directly activating collagen synthesis transcription and stabilizing procollagen mRNA. [15] Along with other properties, Vitamin C is said to have anti-inflammatory and wound healing properties. Therefore, it is of interest to analyse the cyto-protective effects of ascorbic acid on gingival fibroblasts.

## Material and Methods:

## Cell line:

Human Gingival Fibroblast (HGF) was obtained by enzyme digestion from patient undergoing tooth extraction with no clinical history of periodontitis or other oral disease. Written informed consent was collected from patients and ethical approval was obtained from the Institutional Ethical Committee of Saveetha University, Chennai. The tissue was then cut with a sharpened surgical blade and digested with type 1 collagenase and dispase. Cell suspension was cultured in a 25cm2 flask in DMEM: F12 (Gibco, USA) with 20% fetal bovine serum (FBS) and antibiotics (100U/ml pencillin and 100 mg/ml streptomycin) at 37 °C and 5% CO2, for further experiments.

## Isolation of Human Gingival Fibroblast (HGF):

Small pieces of tissues were transferred into Enzyme solution for 1 hr at 37°C. It is vortexed every 30 min to break the tissue. Afterwards, large cell aggregates were removed and Single-cell suspensions were obtained by passing cells through a 70-µM cell strainer. Single-cell suspensions were centrifuged at 1,200 rpm for 5 min at room temperature. Supernatants were carefully pipetted off and pellet was re-suspended in 1 ml proliferation medium (PM). (Note: FBS in proliferation medium terminate enzymatic dissociation). Single-cell suspensions of gingiva were seeded into 25 cm2 culture flask with PM and then incubated at 37°C in 5% CO2. The culture medium was changed every three days until the cell confluency was achieved. Cell suspension was cultured in a 25cm2 flask in DMEM: F12 (Gibco, USA) with 20% fetal bovine serum (FBS) and antibiotics (100U/ml pencillin and 100 mg/ml streptomycin) at 37°C and 5% CO2, for further experiments.

## Chemicals and antibodies:

DMEM medium, 0.25% Trypsin-EDTA solution, sodium bicarbonate solution, bovine serum albumin (BSA), low melting agarose, MTT from Sigma Chemicals Co., St. Louis, USA were used. fetal bovine serum (FBS) and antibiotic/antimycotic solution, DMSO were from Himedia, Sodium phosphate monobasic and dibasic, sodium chloride, sodium hydroxide, sodium carbonate, hydrochloric acid and methanol were purchased from Sisco Research Laboratories (SRL) India.

## Experimental Design:

GROUP I:CONTROL

GROUP II:VITAMIN C

## MTT Assay:

The effect of vitamin Con human gingival fibroblast cells was measured by MTT assay. Briefly, the cells (1 X 105 cells per ml) were seeded in a 96 well micro titer plate (100 µl per well) with replications. Treatment was conducted for 24h with control and experimental group. After 24hrs treatment with control and experimental samples, a 20 µl of 5 mg/ml MTT stock solution was added to each well and incubated for 4 h at 37°C. The obtained formazan crystals were solubilized with DMSO and the absorbance was measured at 570 nm using a microplate reader (Spectra Max M5, Molecular Devices, USA). Cell viability (%) has been shown as a ratio of absorbance (A570) in treated cells to absorbance in control cells (0.1 % DMSO) (A570). The IC50 was calculated as the concentration of sample needed to reduce 50 % of the absorbance in comparison to the DMSO-treated control. Percent cell viability was calculated following the equation:

Cell viability (%) = {A570od of (sample)/A570 od of (control)} x 100

## Statistical analysis:

All data obtained were analyzed by Student's-t-test using MS-Excel, represented as mean ± SD for each group. The results were computed statistically (SPSS/10 Software Package; SPSS Inc., Chicago, IL, USA) using one-way ANOVA. In all tests, the level of statistical significance was set at p<0.05.

## Results:

## Cell viability of Vitamin Con HGF:

**Figure 1:**
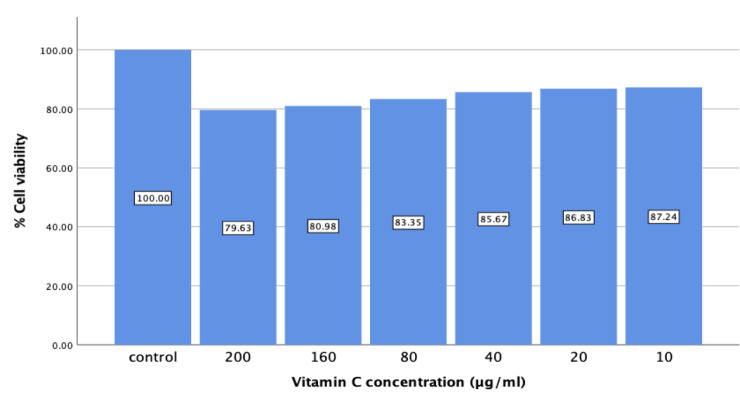
Bar graph represents the mean cell viability of positive control and Vitamin C Group tested by MTT assay for 24 hrs respectively, and absorbance was taken at 570 nm.

The viability assay showed >95% live cells, and no significant (P > 0.05) difference in these values was observed at different concentrations at 24 hrs. The levels of cell proliferation were not significantly different among the control and experimental groups in 24 hrs experimental time-points (p > 0.05). (Figure 1) The cell viability of Positive control and experimental Group (I and II) was tested by MTT assay for 24 hrs respectively and absorbance was taken at 570 nm. Data are shown as means ± SD (n = 3). * compared with the positive control and experimental groups, p < 0.001. Ascorbic acid maintains the cell homeostasis by not disrupting the mitochondrial reductase activity and reducing the mitochondrial stress in the process.

## Cell morphology evaluation:

**Figure 2:**
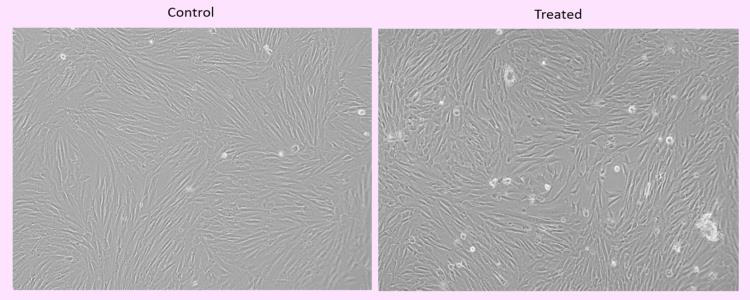
Cell culture of gingival fibroblast at 21 day for placebo and Ascorbic acid

Assessment of cell morphology of HGF cells were treated Vitamin C for 24 h. Images were obtained using an inverted Phase contrast microscope at 10x magnification. (Figure 2)

## Discussion:

In the current investigation, the mitochondrial activity of living cells was measured using the 3-(4,5-dimethylthiazol-2-yl)-2, 5-diphenyl tetrazolium bromide (MTT test) to determine cell viability. [16] These cell culture-based in vitro tests are advantageous since they are quick, easy, inexpensive, and allow for some experimental parameters to be controlled (pH, CO2 concentration, and levels of some molecules). [17,18] This assay measures the cell vitality and cytotoxicity of the drug, the null hypothesis is rejected, and the alternate hypothesis stating that Vitamin C had an non-toxic effect on human gingival fibroblast at any given concentrations.

Previous studies have proven that periodontal disease can lead to an increase in reactive oxygen species that is stimulated by periodontopathic bacteria and can lead to host tissue damage along with bacterial death. [19] It is clear that this proteolytic cascade, which is expressed by multiple collagenolytic MMPs rather than just one in periodontal disorders, is what causes the tissue damage that is a characteristic feature of periodontitis. Tissue damage includes destruction of collagen and reduction in the production of collagen simultaneously. Matrix metalloproteinases or collagenases were high in concentrations in patients with periodontitis was proved by many researchers. MMP-8, MMP-9 and MMP-13 were higher in patients with periodontitis. [20] In inflamed gingival tissue, the majority of proteinases contributes to the degradation of the extracellular matrix and is likely to trigger one another in proteolytic cascades. This was demonstrated to have increased in those with periodontitis.[21]

Ascorbic acid showed cytoprotective effect on skin fibroblasts against the oxidative changes of proteome. Ascorbic acid's antioxidant properties also prevented lipid peroxidation products from changing proteins. [22] The expression of COL1, FN, IL-6, and bFGF, which are connected to fibroblast wound healing activity, is increased by 50 g/ml of ascorbic acid. [23] Expression of genes like CD44 (adhesion gene), TGFB3 COL1A1 and LAMA3 extracellular genes responsible for gingival fibroblast proliferation, migration, adhesion and extracellular matrix have seem to be downregulated in smoking patients. These levels were improved after treatment of ascorbic acid on fibroblasts. [24] Mesenchymal stem cells from the human gingiva (hGMSCs) and endothelial-differentiated (e-hGMSCs) hGMSCs demonstrated elevation of p300 and downregulation of DNA methyltransferase 1 (DNMT1), proteins linked to DNA methylation and histone acetylation, after exposure to LPS-G. In hGMSCs and e-hGMSCs, the combination of AA and LPS-G revealed physiological expression of p300 and DNMT1. Moreover, analysis of reactive oxygen species (ROS) and their intracellular location provided evidence of the inflammatory process sparked by LPS-G. When exposed to AA, the physiological ROS levels were restored. [25] Ascorbic acid had shown to have positive effects on gingival fibroblast by different mechanisms and pathways. Our team has extensive knowledge and research experience that has translate into high quality publications. [26,27, 28,29,30, 31,32,33, 34,35>]

## Conclusion:

The present study estimated the in vitro cell viability of Vitamin Cwith different was assessed by MTT assays that showed pronounced noncytotoxic activity against HGF cells in control and experimental groups. From the MTT results we concluded even at 200µg/ml concentration more than 80% cells are viable, which clearly stated that the above-mentioned drug - Vitamin C is nontoxic and can be used for further experiments to validate for clinical application. This was further confirmed with morphological examination after 24hrs incubation on control and experimental group drugs observed under the phase contrast microscope.

## References

[1] Brookes PS (2004). American Journal of Physiology Cell Physiology..

[2] Wallace DC. (2005). Annu Rev Genet..

[3] Goyal G (2007). Dev Cell..

[4] Nicholls DG, Ferguson SJ (1992). Bioenergetics 2..

[5] Halliwell B, Gutteridge JMC (2015). Free Radicals in Biology and Medicine (3rd ed.).

[6] Brookes P, Darley-Usmar VM (2002). Free Radic Biol Med.

[7] Inoue M (1999). Free Radic Res.

[8] Brookes PS (2002). Free Radic Biol Med,.

[9] Liu X (1996). Cell..

[10] Loeffler M, Kroemer G (2000). Exp Cell Res..

[11] dela Asunción JG (2004). Life Sci..

[12] Chapple I.L, Matthews JB (2007). Periodontology 2000..

[13] Yan Y (2013). Protein Cell..

[14] Traikovich SS (1999). Arch Otorhinol Head Neck Surg..

[15] Farris PK (2009). Cosmetical vitamins: vitamin C. Cosmeceuticals. Procedures in Cosmetic Dermatology. 2nd ed..

[16] Tolosa L (2015). Methods Mol. Biol..

[17] Schweikl H, Schmalz G Eur. (1996). J. Oral Sci..

[18] Nunes YRF (2008). Rev. Arvore.

[19] Kakimoto M (2002). Diabetes.

[20] Gursoy et (2013). Journal of Clinical Periodontology..

[21] Cox S (2005). Oral Diseases.

[22] Gęgotek A (2020). Nutrients..

[23] Chaitrakoonthong T (2020). Int J Dent..

[24] Alyami R (2022). Saudi Dent J..

[25] Pizzicannella Jacopo (2021). Histochemistry and Cell Biology.

[26] Adhinarayanan R (2020). Energy Sources Part A..

[27] Asha P (2022). Environ Res..

[28] Aurtherson PB (2021). Biomass Conversion and Biorefinery.

[29] Johnson J (2020). Hypertens Res..

[30] Mathivadani V. (2020). Acta Virologica.

[31] Ma Y Karunakaran  T (2019). Biotechnol Bioprocess Eng..

[32] Ponnanikajamideen M (2019). Can J Diabetes..

[33] Shanmugam R (2021). Energy Sources Part A..

[34] Thanikodi S (2020). Therm Eng.

[35] Maheswari U (2020). Brazilian Oral Research.

